# Treatment of industrial contaminants with zero-valent iron- and zero-valent aluminium-activated persulfate: a case study with 3,5-dichlorophenol and 2,4-dichloroaniline

**DOI:** 10.3906/kim-1911-60

**Published:** 2021-04-28

**Authors:** Olga KOBA UCUN, Bahareh MONTAZERI, İdil ARSLAN ALATON, Tuğba ÖLMEZ HANCI

**Affiliations:** 1 Department of Environmental Engineering, School of Civil Engineering, İstanbul Technical University, İstanbul Turkey

**Keywords:** 3,5-dichlorophenol, 2,4-dichloroaniline, advanced oxidation, sulfate radicals, persulfate activation with zero-valent metals

## Abstract

Zero-valent iron (ZVI)- and zero-valent aluminium (ZVA)-activated persulfate (PS) oxidation procedure was applied to remove the industrial pollutants 3,5-dichlorophenol (3,5-DCP; 12.27 µM) and 2,4-dichloroaniline (2,4-DCA; 12.34 µM) from aqueous solutions. The effects of PS concentration and pH were investigated to optimize heterogeneous treatment systems. Negligible removals were obtained for both pollutants by individual applications of nanoparticles (1 g/L) and PS (1.00 mM). PS activation with ZVI resulted in 59% (1.00 mM PS; 1 g/L ZVI; pH 5.0; 120 min) and 100% (0.75 mM PS; 1 g/L ZVI; pH 5.0; 80 min) 3,5-DCP and 2,4-DCA removals, respectively. The ZVA/PS treatment system gave rise to only 31% 3,5-DCP (1.00 mM PS; 1 g/L ZVA; pH 3.0; 120 min) and 47% 2,4-DCA (0.25 mM PS; 1 g/L ZVA; pH 3.0; 120 min) removals. The pH decreases from 5.0 to 3.0 and from 3.0 to 1.5 enhanced contaminant removals for ZVI/PS and ZVA/PS treatments, respectively. Pollutant removal rates were in correlation with the consumption rates of the oxidants. Metal ion (Al, Fe) release increased in the presence of PS and with decreasing pH.

## 1. Introduction

Chlorophenols (CPs) and chloroanilines (CAs) are persistent organic pollutants formed during different industrial processes. CPs mostly originate from the pulp paper industry, wood preserves, dyes, drugs, fibers, plastic and leather production and also used as disinfectants and fungicides [1,2]. CAs are associated with herbicide degradation products as well as textile, dye and leather manufacturing processes. 2,4-dichloroaniline (2,4-DCA) is commonly used as an intermediate in the dye manufacturing process [3]. 

Due to their incomplete removal in the natural environment and conventional engineered treatment systems, the abovementioned contaminants are continuously being discharged from wastewater treatment plants to the aquatic environment. Despite the fact that the usage of CPs has decreased recently, their adverse environmental and health effects continue. CPs concentrations in surface water have reached 10 ng/L, while the concentration of CAs in dye manufacturing effluent changes in the range of 7–96 ppb (µg/L) [4,5]. Both pollutants reach the soil environment and degrade to even more harmful intermediates [6,7]. CP concentrations in uncontaminated soil and near sawmill have been reported as 0.001–0.018 mg/kg, and 10,000 mg/kg, respectively [3,8]. 

Both pollutants may lead to detrimental effects in aquatic and soil organisms [9–13]. Moreover, CPs can inhibit the activity of nitrifiers in activated sludge treatment systems [14,15]. Considering all the abovementioned issues, some CPs were included in the EU list of priority substances [16]. The U.S. Environmental Protection Agency (USEPA) has recommended water quality criteria in the range of 0.04–1800 µg/L for different CPs [17]. As such, their efficient removal by advanced treatment methods remains a prime issue and challenging task.

In the last three decades, advanced oxidation processes (AOPs) based on in situ formation of reactive species (free radicals) have been applied for the efficient degradation and detoxification of organic and inorganic compounds including industrial micropollutants [1,2,18–23]. The most frequently used AOPs are enhanced ozonation [24], photocatalysis [2,18], the Fenton’s reagent [25], the photo-Fenton processes [18,26], electrochemical degradation [27], sonolysis [28], microwave irradiation [29], wet air oxidation [30], electrohydraulic discharge (plasma) [31] and their combinations [32]. AOPs basically rely on the formation of the hydroxyl radicals (HO·) besides other free radicals. Compared to sulfate radicals [SO_4_^·-^, E^0^ = 2.5–3.1 V vs. standard hydrogen electrode (SHE)]; half-life (t_1/2_ = 30–40 µs) HO· (E^0^ = 1.89–2.72 V vs. SHE; t_1/2_ = 10^-3^ µs) have a lower reduction potential but longer half-life [33–35]. Consequently, SO_4_^·-^-based AOPs could be an interesting alternative for water and wastewater treatment applications [36].

Usually, SO_4_^·-^ can be produced by activation of persulfate (PS) and peroxymonosulfate (PMS) with ultraviolet (UV) irradiation [37], heat [21], ascorbic acid [22], metal oxides [20], transition metal ions (Me^n+^) [38] and zero-valent metals [39]. Particularly the use of zero-valent iron (ZVI) and aluminium (ZVA) has recently received great attention due to their unique surface properties, high abundance in the earth’s crust and high reactivity. ZVA (E^0^ = –1.67 V vs. SHE) has a higher oxidation potential than ZVI (E^0^ = –0.44 V vs. SHE), providing a stronger thermodynamic driving force for electron transfer [23]. Apparently, ZVI is a better candidate in terms of PS activation than Fe^2+^ since the continuous and slow release of Fe^2+^ ions from heterogeneous ZVI (FE^0^) provides more efficient PS activation and SO_4_^·-^ generation compared to the homogeneous Fenton-like treatment system [40]. The PS activation mechanism using ZVI and ZVA materials has been described in former works [41–48]. Accordingly, under acidic pH conditions (≤ 5.0) in the presence of dissolved oxygen (O_2_), SO_4_^·-^ formation occurs as shown below.

PS activation with ZVA (ZVA/PS):

2Al^0^ + S_2_O_8_^2−^ + 6H+ + 1.5O2 → 2Al3+ + 2SO_4_^•-^ + 3H_2_O (1)

PS activation with ZVI (ZVI/PS):

FE^0^ + S_2_O_8_^2−^ → Fe^2+^ + 2SO_4_^2-^ (2)

Fe^2+^ + S_2_O_8_^2−^ → Fe^3+^ + 2SO_4_^2-^ + SO_4_^•-^ (3)

FE^0^ + 2S_2_O_8_^2−^ → Fe^2+^+ 2SO_4_^2-^ + 2SO_4_^•-^ (4)

Fe^2+^_(surf)_ + S_2_O_8_^2−^ → Fe^3+^_(surf)_ + SO_4_^2-^ + SO_4_^•-^ (5)

However, major drawbacks of Fenton-like reactions can be listed as the i) production of sludge waste, ii) release of toxic metals and iii) requirement of an acidic reaction pH (2 < pH < 5) that would increase operational maintenance costs and impacts on the environment. To the best of our knowledge, limited information on the alternative treatment methods for 3,5-DCP and 2,4-DCA in liquid matrices is available [49,50]. The current experimental study aimed at examining the degradation of aqueous 3,5-DCP and 2,4-DCA with the heterogeneous ZVI/PS and ZVA/PS treatment systems. The present study focused on the effect of PS concentration and pH on the treatment systems. The treatment performances were comparatively evaluated in terms of model pollutant removal, oxidant (PS) consumption and metal ion (Fe, Al) release rates. 

## 2. Materials and methods

### 2.1. Materials

3,5-DCP (formula: C_6_H_4_Cl_2_O; molecular weight: 163 g/mol; CAS: 591-35-5) and 2,4-DCA (formula: C_6_H_5_Cl_2_N; molecular weight: 162 g/mol; CAS: 554-00-7) were purchased from Sigma-Aldrich (Hamburg, Germany). Solubility in water was reported as <1 g/L at 20 °C and 23 °C for 3,5-DCP and 2,4-DCA, respectively [51]. Potassium persulfate (formula: K_2_S_2_O_8_; molecular weight: 270 g/mol; CAS: 7727-21-1; Sigma-Aldrich), methanol and LC-MS grade water were supplied by Merck (Darmstadt, Germany). Commercial nanoscale ZVI (average particle size 50 nm; BET surface area 20–25 m^2^/g; purity > 99.5%) was purchased from Nanofer Star (Nano Iron, Židlochovice, Czech Republic). High purity (>99.5%) ZVA nanoparticles (average particle size = 100 nm; specific surface area = 10–20 m^2^/g) were obtained from US Research Nanomaterials, Inc. (Houston, USA). 

### 2.2. ZVI/PS and ZVA/PS treatment systems 

A working concentration of 2 mg/L was selected for the model micropollutants to ensure an accurate kinetic investigation and analytical assessment. It should be noted here that the actual concentration of the selected model pollutants is by far lower; typically, in the ng/L – µg/L range [4,5]. All experiments were carried out in 500 mL capacity borosilicate glass beakers under continuous stirring at 150 rpm to provide saturated air oxygen and proper distribution of zero-valent metal nanoparticles in the reaction solution. 

Prior to each experiment, the initial pH of the reaction solutions (2 mg/L) was adjusted to the desired value (pH = 3.0 and 5.0 for ZVI/PS; pH = 1.5 and 3.0 for ZVA/PS) by adding 1–4 N H_2_SO_4_ solutions. These pH values were chosen in accordance with Fenton/Fenton-like chemistry, which is limited to acidic reaction conditions (pH ≤ 4) [52]. Thereafter, 1 g/L of ZVI or ZVA was added to the reaction mixture. Finally, PS was put in the solution at a specified initial concentration (0.00–1.00 mM PS). Experimental conditions such as PS and zero-valent metal concentrations were selected based on our preliminary optimization results and referring to previously published related studies [39,41–44,48]. 

Samples were taken out at regular time intervals for up to 120 min during the experiments. All samples were immediately filtered through 0.22 µm PVDF syringe filters (GVS, USA) to remove zero-valent metal nanoparticles. In addition, the pH of the ZVI/PS samples was increased to ≈ 7.0–7.5 with NaOH solution to remove dissolved iron in the form of ferric hydroxide flocs as well as to stop Fenton/Fenton-like bulk reactions. Each experiment was conducted in duplicate and whenever unusual or erroneous results were obtained, the experiment was repeated. 

### 2.3. Analytical procedure and instrumental analysis

The analysis of target compounds was carried via HPLC (Agilent 1100 Series; Agilent Technologies, Santa Clara, CA, USA) that was coupled with a diode array detector (DAD, G1315A, Agilent Series). Measurements were done at wavelength of 285 and 240 nm for 3,5-DCP and 2,4-DCA, respectively. A Nova-Pak C18 (3.9 mm × 150 mm, 4 µm, Waters Corporation, Milford, MA, USA) reversed-phase column was used. The mobile phase consisted of 70% methanol and 30% ultrapure water. The flow rate and temperature of the column were set as 1.0 mL/min and 25°C, respectively. The injection volume was 100 µL. Analytical methods were validated for a concentration range of 0.0625–2 mg/L. Six-point calibration curves showed good linearity (R^2^) with correlation coefficients of 0.9998 and 0.9996 obtained for 3,5-DCP and 2,4-DCA, respectively. Detection and quantification limits were established as 0.06 and 0.21 mg/L for 3,5-DCP and 0.03 and 0.12 mg/L for 2,4-DCA, respectively. An Orion (USA) 720A+ model pH-meter was used for all pH measurements. A Jenway 6300 model spectrophotometer (Staffordshire, UK) was employed for colorimetric, residual PS analyses [53]. Fe and Al releases were monitored on a Perkin-Elmer ICP-MS (PerkinElmer, Inc., Waltham, MA, USA) [54].

## 3. Results and discussion

### 3.1. Control experiments: PS, ZVI/air/H^+^ and ZVA/air/H^+^

**Figure 1:**
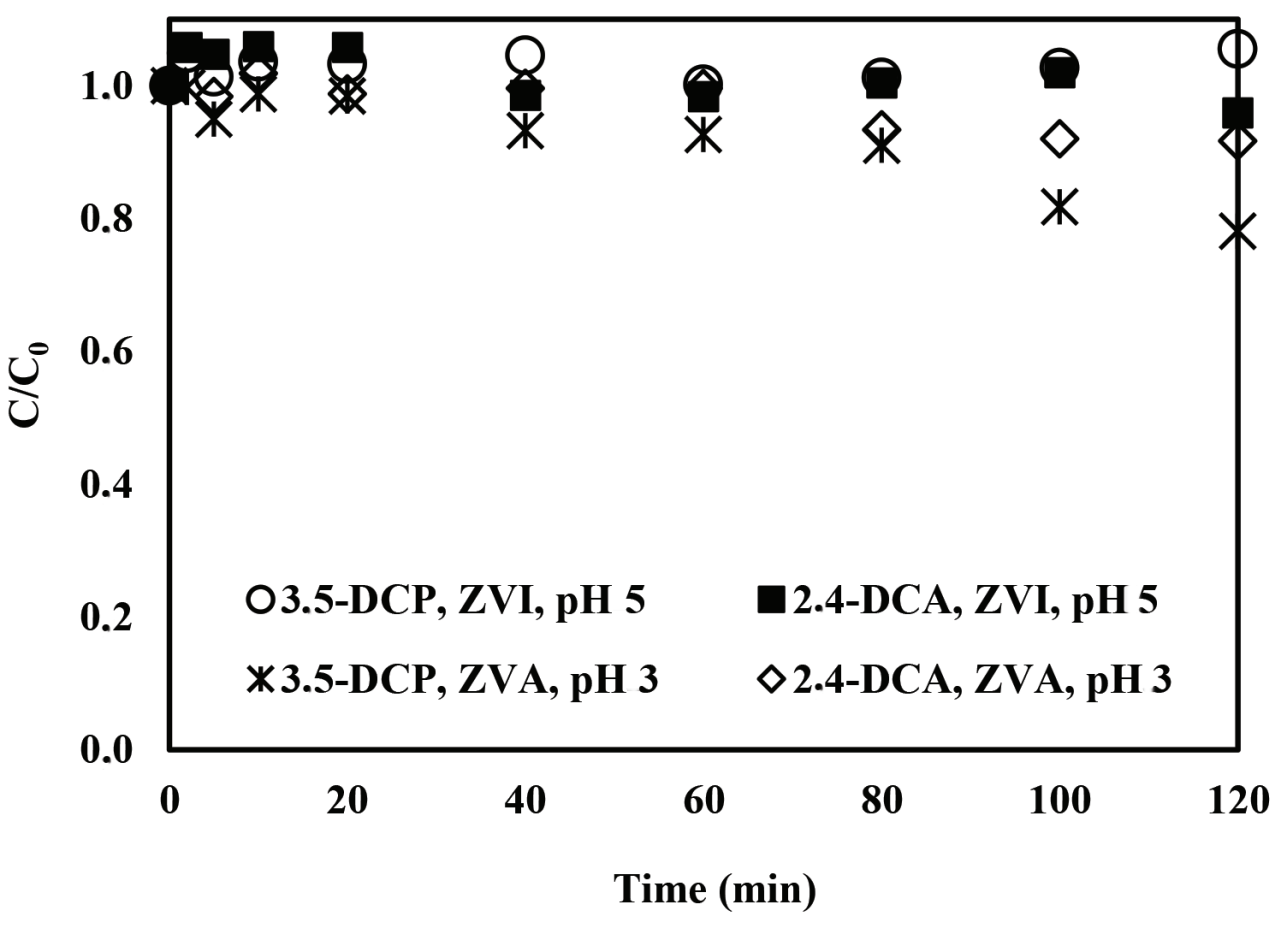
Normalized 3,5-DCP and 2,4-DCA abatements obtained during the application of ZVA/air/H^+^ and ZVI/air/H^+^ treatment systems. Experimental conditions: 3,5-DCP and 2,4-DCA = 2.0 mg/L, ZVI and ZVA = 1 g/L.

In the first part of the study, control experiments were conducted (2 mg/L aqueous pollutant; 1.00 mM PS only; 1 g/L ZVI only; or 1 g/L ZVA only) to examine the effect of ZVI on PS activation. Results indicated that without PS activation with zero-valent metals no 3,5-DCP and 2,4-DCA removals occurred (data not shown). Moreover, ZVI/air/H^+^ at pH 5.0 and ZVA/air/H^+^ treatments at pH 3.0 resulted in poor 3,5-DCP and 2,4-DCA removals (0 - 22%). As may be seen in Figure 1, neither PS without activation nor zero-valent metal nanoparticles in the absence of PS were effective in terms of 3,5-DCP and 2,4-DCA degradation. Limited pollutant removals have already been evidenced in previous studies without oxidant activation [41–44]. 

Similarly, in a study of Bokare and Choi [23], 4-chlorophenol (4-CP) degradation could not be achieved with ZVI/air/H^+^ and ZVA/air/H^+^ treatments (reaction conditions: 4-CP = 0.1 mM, ZVI and ZVA = 1 g/L, pH = 2.5) even after 10 h revealing that is important for fast and efficient oxidation of pollutants PS activation. All removal efficiencies for the control experiments are shown in supplementary materials (Table S1). 

### 3.2. The ZVI/PS treatment system

#### 3.2.1. Effect of PS concentration 

**Figure 2:**
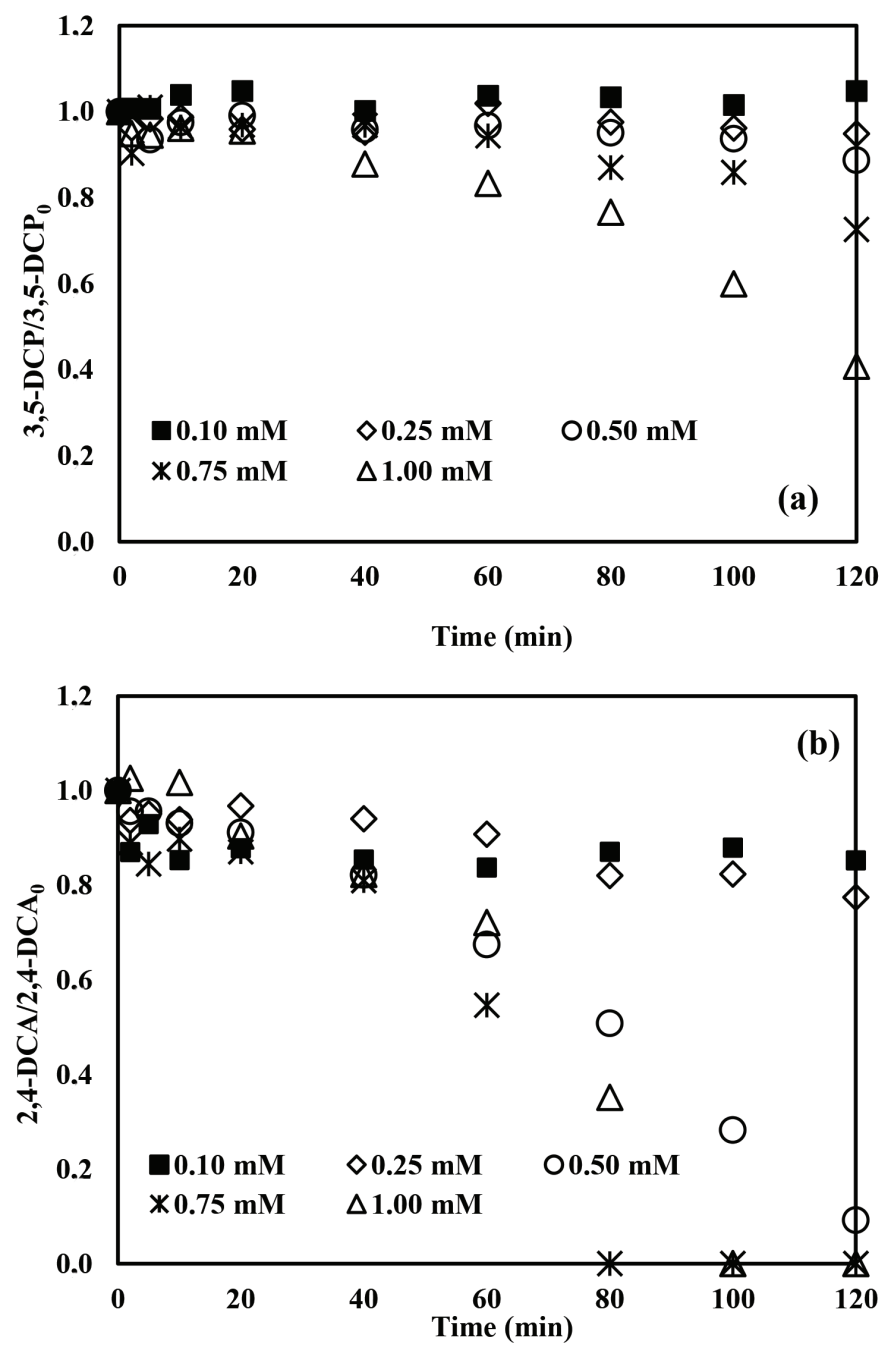
Effect of initial PS concentration on normalized 3,5-DCP (a) and 2,4-DCA (b) abatements by application of the ZVI/PS treatment system. Experimental conditions: 3,5-DCP and 2,4-DCA = 2 mg/L, ZVI = 1 g/L, pH = 5.0.

**Figure 3:**
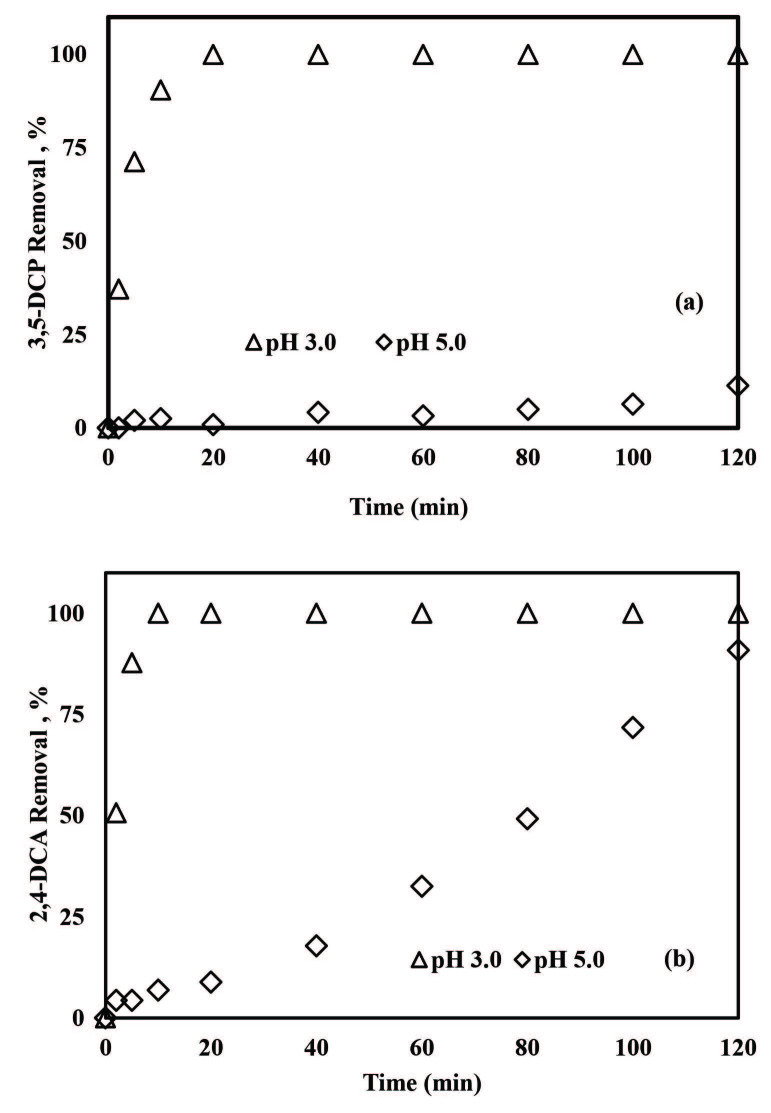
The effect of pH on 3,5-DCP (a) and 2,4-DCA (b) removals with the ZVI/PS treatment system. Experimental conditions: 3,5-DCP and 2,4-DCA = 2 mg/L, PS = 0.50 mM, ZVI = 1.0 g/L.

Firstly, PS concentrations in the range of 0.10–1.00 mM were tested for ZVI/PS treatment (Figure 2). An induction phase of 40 min was evidenced for both pollutants at all examined PS concentrations (Figure 2a). According to Figure 2a, 3,5-DCP removal was insignificant ( < 5%) for 0.1 and 0.25 mM PS but started to increase appreciably at elevated PS concentrations. After 120 min treatment, 3,5-DCP removals were obtained as 11%, 27%, and 59% for 0.5, 0.75 and 1.00 mM PS, respectively.

Figure 2b displays 2,4-DCA abatement profiles for ZVI/PS treatment. As can be seen from Figure 2b, the degradation of 2,4-DCA was clearly enhanced by increasing the initial PS concentration from 0.10 to 0.75 mM. 2,4-DCA removal efficiencies obtained after 120 min treatment in the presence of 0.10, 0.25 and 0.50 mM PS were 15%, 23% and 91%, respectively. Complete 2,4-DCA degradation was achieved after 80 min treatment with 0.75 mM PS. On the other hand, when the initial PS concentration increased from 0.75 mM to 1.00 mM, the time required for complete 2,4-DCA removal increased from 80 min to 100 min. This effect might be due to “SO_4_^•-^ scavenging” reactions in the presence of excessive PS and/or Fe^2+^ concentrations or radical recombination reactions as given in Eqs. 6–8 [42,43,45,55,56]:

S_2_O_8_^2−^ + SO_4_^•-^ → S_2_O_8_^•−^ + SO_4_^2-^ *k *= 6.1 × 10^5^ M^-1^s^-1^ (6)

Fe^2+^ + SO_4_^•-^ → Fe^3+^ + SO_4_^2-^ *k *= 3 × 10^8^ M^-1^s^-1^ (7)

SO_4_^•-^ + SO_4_^•-^ → S_2_O_8_^2−^ *k *= 4 × 10^8^ M^-1^s^-1^ (8)

In another recent study, a PS increment from 0.787 mM to 1.050 mM resulted in a decrease in the reaction rate constant from 0.0753 to 0.0182 min^-1^ during PS/ZVI treatment of bentazon [45]. Hayat et al. [46] investigated the degradation of 30 ppm imidacloprid with ZVI/PS where it was found that increasing the PS concentration from 2.5 mM to 10 mM decreased the pollutant removal rates from 88 to 59% after 180 min treatment [46]. In the present study, the ZVI/PS treatment system could not provide complete elimination of 3,5-DCP at pH 5.0 but complete 2,4-DCA removal was achieved after 80 min treatment with 0.75 mM PS.

#### 3.2.2. Effect of pH 

It has already been demonstrated that pH is a critical parameter for micropollutants removal by the ZVI-activated PS oxidation system [42,43,45,46,57–59]. In the present study, pH values of 3.0 and 5.0 were tested with the ZVI/PS treatment system in the presence of 0.50 mM PS. From Figure 3a it is apparent that 3,5-DCP removals were enhanced when the initial solution pH was decreased from 5.0 to 3.0 for ZVI/PS treatment. The overall 3,5-DCP removal efficiency was obtained as 11% at pH 5.0 after 120 min treatment, whereas complete 3,5-DCP removal was attained at pH 3.0 after 20 min treatment. Ninety-one percent of of 2,4-DCA removal was achieved after 120 min with ZVI/PS treatment at pH 5.0 (Figure 3b). Lowering the pH from 5.0 to 3.0 accelerated the 2,4-DCA degradation rate appreciably which resulted in 100% removal after 10 min treatment. The results of the present study are in agreement with previous findings where a pH of 3 was recommended to achieve the highest bentazon degradation with the ZVI/PS treatment system over a wide pH range (3–11) [45]. 

The optimum pH of the Fenton and Fenton-like reactions was reported as 3.0 ± 0.2 [57]. The pH of the medium affects the speciation of dissolved metal species as well as the activity and decomposition rate of peroxides [57]. Based on the data obtained in this study, it could be concluded that the acidic reaction conditions should be favored to produce more ferrous ions (Fe^2+^) that play a critical role in SO_4_^•-^ production [45]. It should be reasonable to emphasize here that the surface properties of ZVI (the speciation of iron oxides/hydroxides on the ZVI surface) might have been subjected to change during the ZVI/oxidant treatment systems depending on the pH value [58,59].

#### 3.2.3. PS consumption 

**Figure 4:**
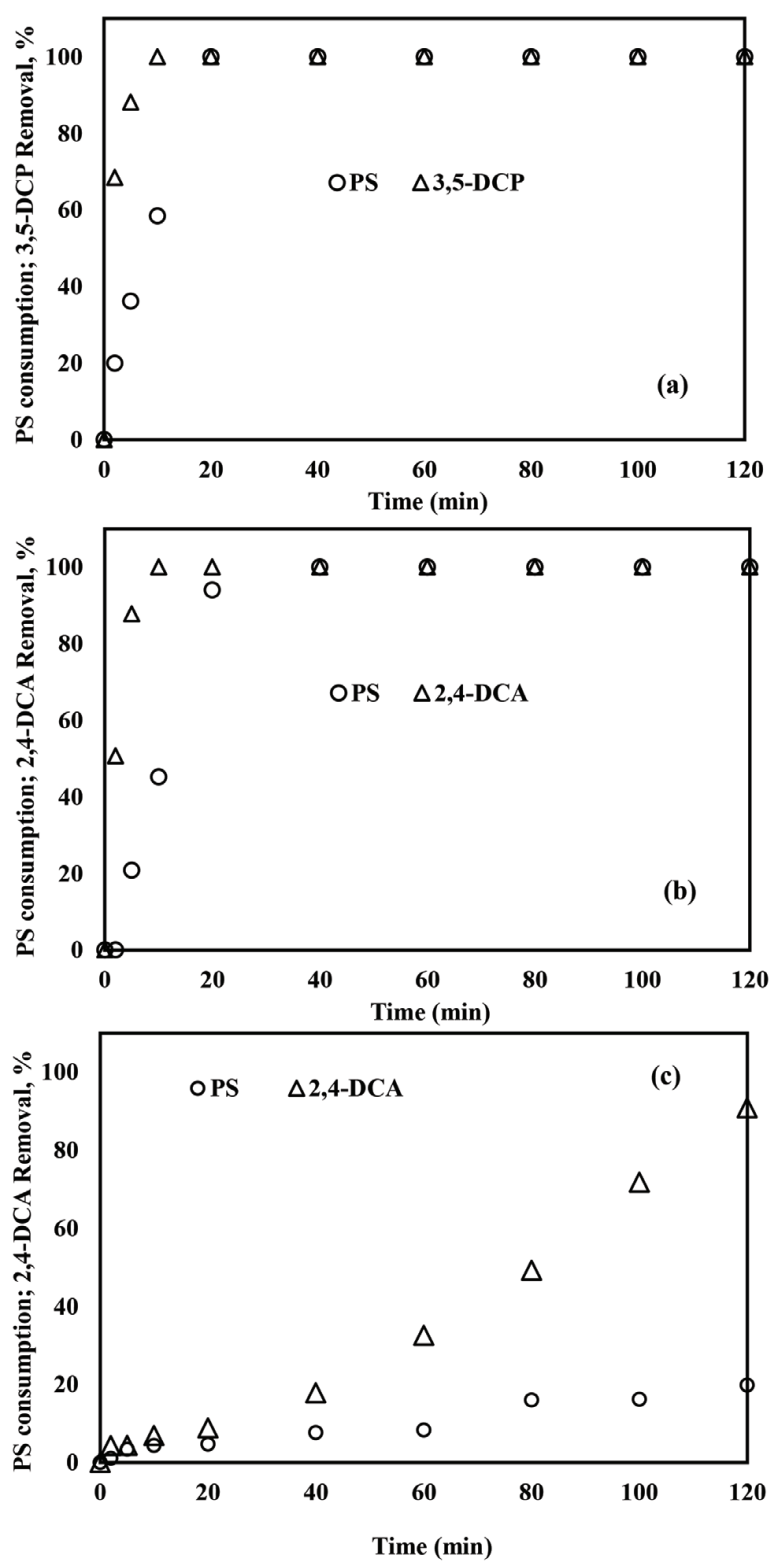
PS consumptions during removals of 3,5-DCP at pH 3.0 (a), 2,4-DCA at pH 3.0 (b) and 2,4-DCA at pH 5.0 (c) with the ZVI/PS treatment system. Experimental conditions: 3,5-DCP and 2,4-DCA = 2 mg/L, PS = 0.50 mM, ZVI = 1 g/L.

In order to question whether PS was successfully activated and then consumed by ZVI for 3,5-DCP and 2,4-DCA degradation, PS concentration was followed during ZVI/PS treatments with 0.50 mM PS at pH 3.0 and 5.0. Complete PS consumption was obtained for both pollutants at pH 3.0 (see Figures 4a and 4b). PS consumptions were completed in 20 and 40 min at pH 3.0 for 3,5-DCP (Figure 4a) and 2,4-DCA (Figure 4b), respectively. It is worth mentioning that complete degradation of 3,5-DCP and 2,4-DCA was obtained after 10 and 30 min when PS was still present in the reaction solution. However, PS consumption for 2,4-DCA at pH 5.0 (Figure 4c) was relatively slow with a low efficiency by 31% after 120 min treatment. The poor PS consumption reflected the slow abatement of 2,4-DCA at pH 5.0 (Figure 4c). 

The specific oxidation efficiencies (i.e. SOE values; standing for mass of target pollutant removed per mass of oxidant consumed) were calculated for the ZVI/PS treatment systems. The SOE values obtained for ZVI/PS (PS = 0.50 mM) at pH 3.0 and 5.0 were 0.022 and 0.023 mg/mg for 3,5-DCP, and 0.023 and 0.104 mg/mg for 2,4-DCA, respectively. The initial pH decrease from 5.0 to 3.0 decreased the SOE values for the ZVI/PS system and almost identical SOE values were obtained for the micropollutants at pH 3.0. 

All removal efficiencies for the ZVI/PS treatment system are exhibited in the supplementary materials (Tables S2 and S3). 

#### 3.2.4. Fe release

In order to evaluate the efficiency of PS activation by ZVI at varying pH’s, dissolved/released Fe concentrations were measured after 120 min treatment of the selected model pollutants. As can be seen from Table 1, the ZVI/air/H+ treatment system resulted in only 0.51 mg/L and 0.22 mg/L Fe release at pH 5.0 for 3,5-DCP and 2,4-DCA, respectively. These data indicated that the Fenton-like reaction was not effective in the absence of PS oxidant. Upon 0.50 mM PS addition, the Fe concentration in the reaction solution increased dramatically from 0 to 22 mg/L at the end of 3,5-DCP treatment at pH 5.0. However, Fe release was still low and limited to 0.24 mg/L during 2,4-DCA treatment under otherwise identical reaction conditions. A similar increase in released Fe concentrations upon PS addition was also evidenced by Dogan et al. [39] in a study on bisphenol A (BPA) degradation with ZVI/air/H^+^ and ZVI/PS treatments. In the presence of PS, Fe release increased from 25 to 200 mg/L (for BPA = 20 mg/L, PS = 2.5 mM, ZVI = 1 g/L and pH 3.0) [39]. On the other hand, a pH decrease from 5.0 to 3.0 significantly enhanced the dissolution of Fe ions for both pollutants (Table 1). The overall released Fe concentrations were 12 and 50 mg/L for 2,4-DCA and 3,5-DCP, respectively. Fe release was positively correlated with PS consumptions and pollutant removals during ZVI/PS treatment of 3,5-DCP and 2,4-DCA under acidic pH conditions.

**Table 1:** Fe concentrations obtained after 120 min treatment of 3,5-DCP and 2,4-DCA with ZVI/air/H+ and ZVI/PS systems. Experimental conditions: 3,5-DCP and 2,4-DCA = 2 mg/L, PS = 0.50 mM, ZVI = 1 g/L.

Pollutant	Process	Total Fe (mg/L)
3,5-DCP	ZVI, pH 5.0	0.51
	ZVI/PS, pH 5.0	22
	ZVI/PS, pH 3.0	50
2,4-DCA	ZVI, pH 5.0	0.22
	ZVI/PS, pH 5.0	0.24
	ZVI/PS, pH 3.0	12

### 3.3. The ZVA/PS treatment system

#### 3.3.1. Effect of PS concentration 

**Figure 5:**
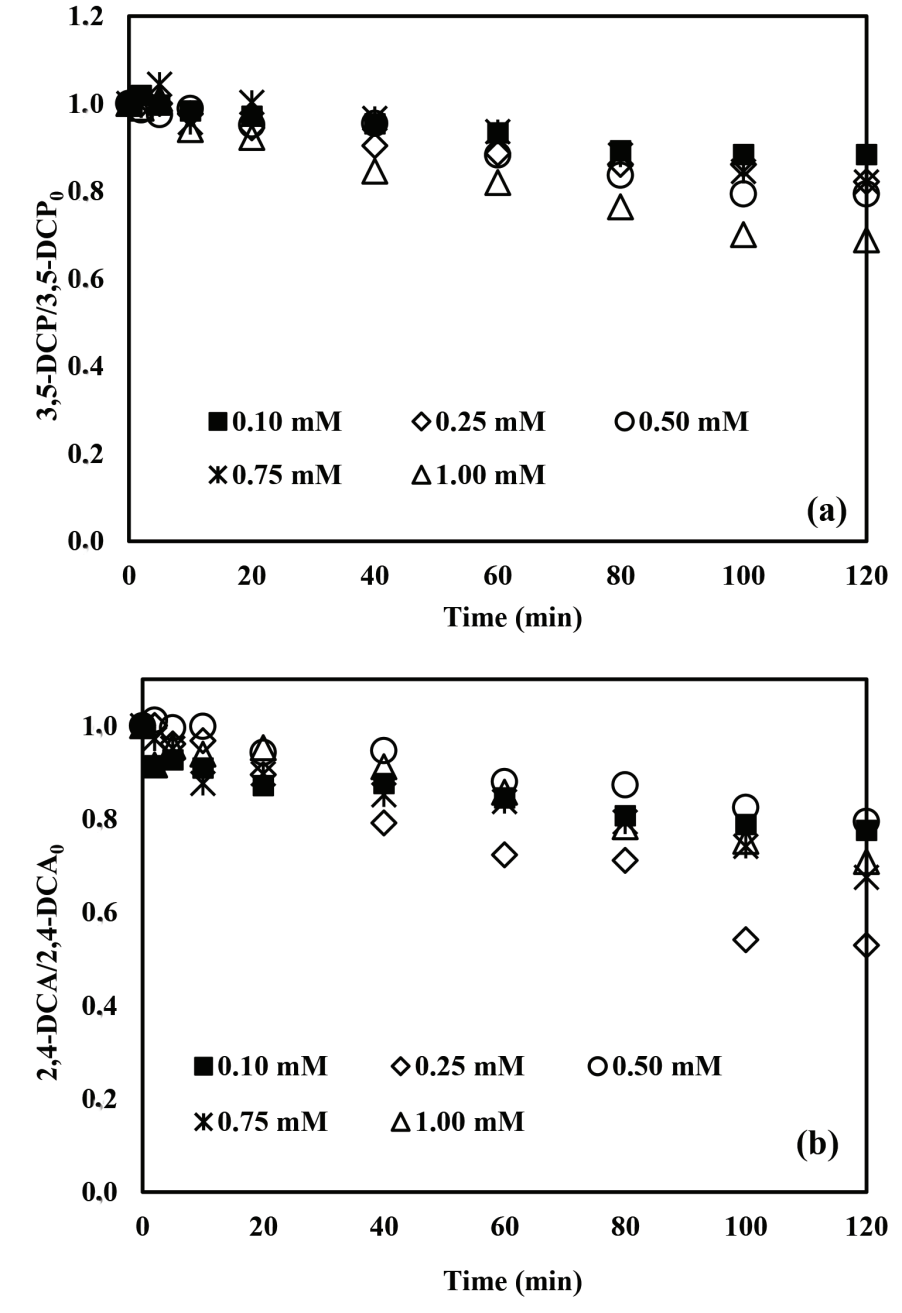
Effects of initial PS concentration on normalized 3,5- DCP (a) and 2,4-DCA (b) abatements during application of the ZVA/PS treatment system. Experimental conditions: 3,5-DCP and 2,4-DCA = 2 mg/L, ZVA = 1 g/L, pH = 3.0.

Careful optimization of the oxidant concentration in Fenton-like reactions is essential achieving full degradation of the target pollutants [41,44,47,48]. The effect of PS concentration on 3,5-DCP and 2,4-DCA removals with the ZVA/PS treatment process was investigated in the PS concentration range 0.10–1.00 mM at pH 3.0. The highest 3,5-DCP abatement was found as 31% in the presence of 1.00 mM PS and in the PS concentration range 0.10–0.75 mM 3,5-DCP removal did not exceed 21% (Figure 5a). On the other hand, the highest 2,4-DCA degradation (47%) was achieved at the initial PS concentration of 0.25 mM after 120 min treatment (Figure 5b). Further increasing of the initial PS concentration did not enhance 2,4-DCA degradation rates. For all other tested PS concentrations, overall 2,4-DCA removals remained in the range of 22%–33%. 

In the study by Ren et al. [47] on trichloroethylene (0.20 mM in distilled water) removal with ZVA/PS in the presence of 2–20 mM PS, the trichloroethylene degradation rate coefficient was found to increase from 0.0076 min^-1^ to 0.0317 min^-1^ with increasing the PS concentration from 2 to 15 mM and decreased with further increase of the PS concentration from 15 to 20 mM to 0.0307 min^-1^ [47]. These results support the presence of a pollutant-specific optimum PS concentration (in the present case 0.25 mM for 2,4-DCA) for ZVA/PS treatment as a consequence of PS overdose that might trigger radical self-quenching and recombination reactions as discussed before and shown by Eqs. 6 and 8. 

In the present study, ZVA/PS treatment of aqueous 3,5-DCP and 2,4-DCA resulted in poor removal efficiencies. This finding is in contrast with previous works where complete degradation of the micropollutants bisphenol A and iopamidol was achieved under similar reaction conditions [41,48]. 

#### 3.3.2. Effect of pH 

ZVA nanoparticles are known to be very reactive and might be easily covered with a hydrated alumina layer (Al2O3 + Al(OH)3) [23,48]. Former studies have demonstrated that acid washing and/or very acidic pH conditions were applied in ZVA/PS treatment to enhance bulk Fenton/Fenton-like reactions [23,41,47,48]. Apparently, the pH of the reaction solution plays a crucial role in the degradation of micropollutants by ZVA/oxidant treatment systems.

**Figure 6:**
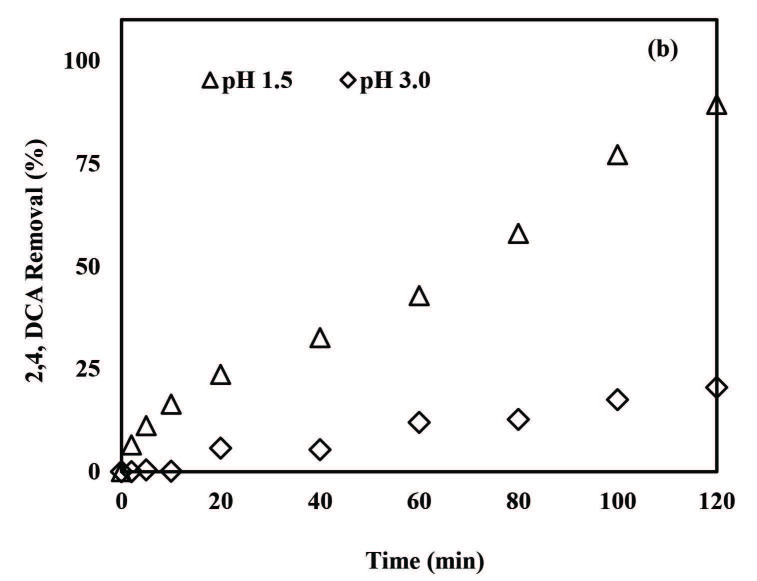
Effect of pH on the removal of 3,5-DCP (a) and 2,4-DCA (b) by the ZVA/PS treatment system. Experimental conditions: 3,5-DCP and 2,4-DCA = 2 mg/L, PS = 0.50 mM, ZVA = 1 g/L.

In order to investigate the effect of pH on 3,5-DCP and 2,4-DCA degradation, experiments were run at pH = 1.5 and 3.0 in the presence of 0.50 mM PS and 1 g/L ZVA. The effect of initial pH on pollutant removal rates is depicted in Figure 6. Removal efficiencies increased from 20% to 77% and 89% for 3,5-DCP (Figure 6a) and 2,4-DCA (Figure 6b), respectively, when the pH was decreased from 3.0 to 1.5. However, complete pollutant removal could not be achieved with ZVA/PS treatment. It was noticed that the pH increased slightly during treatment (from 1.5 to 2.0 and from 3.0 to 3.4) which may instigate the corrosion of ZVA nanoparticles Al oxide and hydroxide layers on their surface [23] (Eq. 9);

(9)2Al0+32O2+3H2O→2Al3++6OH-

However, Bokare and Choi [23] postulated that so long as the solution pH is kept below 4 during the reaction, ZVA dissolution and hence oxidation processes continue.

#### 3.3.3. PS consumption

**Figure 7:**
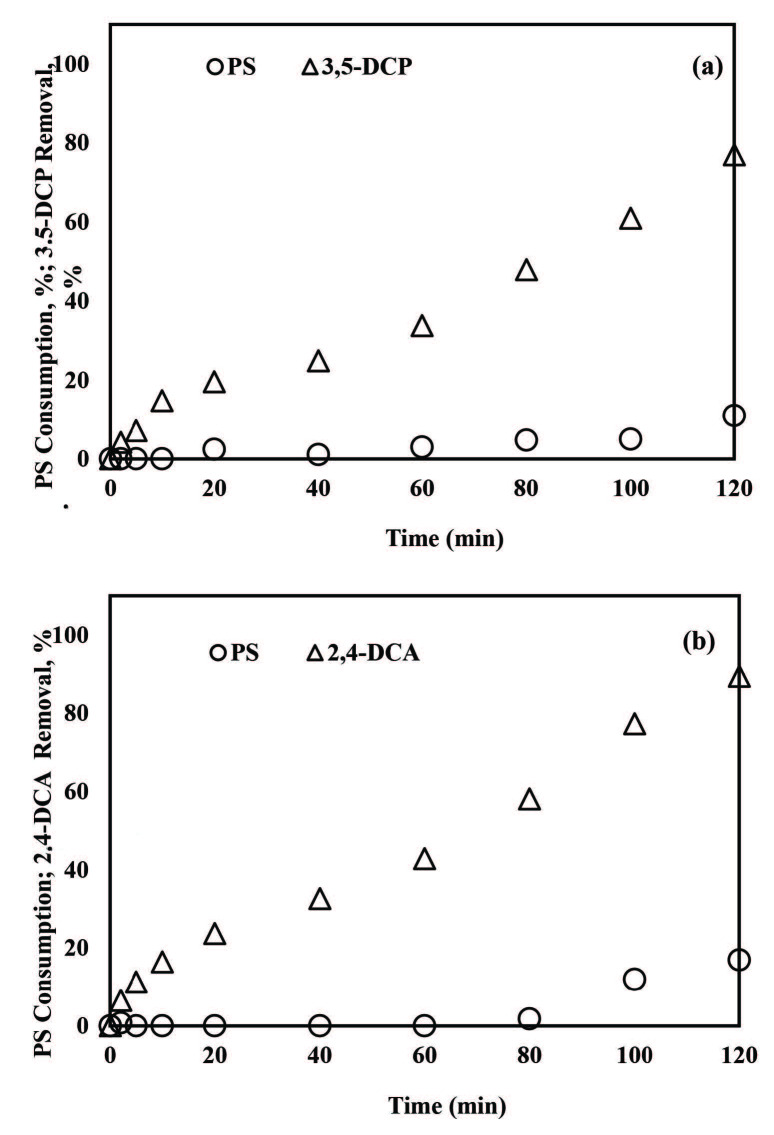
PS consumptions during removals of 3,5-DCP (a) and 2,4-DCA (b) by the ZVA/PS system. Experimental conditions: 3,5-DCP and 2,4-DCA = 2 mg/L, PS = 0.50 mM, ZVA = 1 g/L,pH = 1.5.

Residual PS concentrations were also recorded during the degradation of 3,5-DCP and 2,4-DCA within the ZVA/PS treatment system at pH 1.5 and the results were presented in Figure 7 together with their removal rates. An overall PS consumption by 11% and 17% for 3,5-DCP (Figure 7a) and 2,4-DCA (Figure 7b), respectively, was evident during ZVA/PS treatment. Thus, it might be concluded that inefficient pollutant removal is related to poor oxidant consumption, which was also seen for ZVI/PS treatment at pH 5.0 (Figure 4c). The SOE values were determined as 0.165 mg 3,5-DCP/mg PS and 0.124 mg 2,4-DCA/mg PS which were accompanied by 77% of 3,5-DCP and 89% 2,4-DCA removals, respectively. The SOE values obtained for ZVA/PS treatments were higher than those calculated for ZVI/PS treatment at pH 3.0 under otherwise identical reaction conditions.

All removal efficiencies for the ZVA/PS treatment system are displayed in the supplementary materials (Tables S4 and S5). 

#### 3.3.4. Al release

Al release was monitored during 3,5-DCP and 2,4-DCA degradation with the ZVA/air/H+ and ZVA/PS treatment systems. Al concentrations obtained after the treatments for 120 min at pH 1.5 and 3.0 were presented in Table 2. As can be seen from Table 2, Al release patterns of the pollutants were similar for ZVA/air/H+ treatment at pH 3.0. Surprisingly, PS addition to the reaction solution at pH 3.0 did not induce Al release. These findings presumably indicate that due to rapid corrosion and surface coverage of ZVA at pH = 3.0, poor model pollutant removals were achieved at pH = 3.0 [23,41,47,48]. In another study, 4 mg/L Al release was reported for Triton X-45 degradation with ZVA/PS under practically identical reaction conditions [60]. Al release rates increased by one order of magnitude when the reaction pH was decreased from 3.0 to 1.5. 17 and 20 mg/L Al release occurred after 120 min ZVA/PS treatment of 3,5-DCP and 2,4-DCA, respectively. It can be inferred from these data that Al release and PS consumption may be correlated with 3,5-DCP and 2,4-DCA removals when all the parameters were enhanced for ZVA/PS treatment at the lowest studied pH (= 1.5). 

**Table 2:** Al concentrations obtained after 120 min treatment of 3,5-DCP and 2,4-DCA with ZVA/air/H+ and ZVA/PS systems. Experimental conditions: 3,5-DCP and 2,4-DCA = 2 mg/L, PS = 0.50 mM, ZVA = 1 g/L.

Pollutant	Process	Total Al (mg/L)
3,5-DCP	ZVA, pH 3.0	4.2
	ZVA/PS, pH 3.0	3.9
	ZVA/PS, pH 1.5	17
2,4-DCA	ZVA, pH 3.0	4.5
	ZVA/PS, pH 3.0	4.5
	ZVA/PS, pH 1.5	20

## 4. Conclusion

The activation of PS with ZVI and ZVA nanoparticles was comparatively investigated for the degradation of two commercially important industrial pollutants; namely 3,5-DCP and 2,4-DCA. The effects of initial PS concentration and pH were examined on the basis of 3,5-DCP and 2,4-DCA removals, PS consumptions and metal ion (Fe, Al) release rates. The following conclusions could be drawn from this study;

· PS only, ZVI/air/H+ and ZVA/air/H+ treatments were found inefficient for 3,5-DCP and 2,4-DCA oxidation. 

· ZVI/PS at pH 5.0 resulted in a maximum removal of 59% (1.00 mM PS; 1 g/L ZVI; t = 120 min) and 100% (0.75 mM PS; 1 g/L ZVI; t = 80 min) for 3,5-DCP and 2,4-DCA, respectively.

· A pH decrease from 5.0 to 3.0 for ZVI/PS treatment led to complete removal for both model pollutants at a lower initial PS concentration (0.50 mM) after 20 and 10 min for 3,5-DCP and 2,4-DCA, respectively.

· Fast pollutant removals were accompanied with quick PS consumptions during ZVI/PS treatments. PS was completely consumed after 20 min and 40 min for 3,5-DCP and 2,4-DCA, respectively, at pH 3.0.

· Fe release rates for all processes were correlated with the pollutant removal rates. Fe concentrations were found significantly higher for 3,5-DCP than for 2,4-DCA in all the treatments. 

· The ZVA/PS treatment system at pH 3.0 exhibited 31% (1.00 mM PS; 1 g/L ZVA) and 47% (0.25 mM PS; 1 g/L ZVA) removals for 3,5-DCP and 2,4-DCA, respectively following the treatment of 120 min. 

· The pH decrease from 3.0 to 1.5 improved the micropollutant abatement rates up to 77 and 89% for 3,5-DCP and 2,4-DCA, respectively (0.50 mM PS; 1 g/L ZVA; t = 120 min) for the ZVA/PS treatment process. 

· Only 11% and 17% of the initially added PS (0.50 mM) was consumed at pH 1.5 during ZVA/PS treatment of 3,5-DCP and 2,4-DCA, respectively. 

· Al release data revealed that ZVA corrosion in the presence of PS and at pH 1.5 was more severe for both studied pollutants.

In should be kept in mind that for a better treatment performance more acidic pH conditions (pH 2.0–3.0 for ZVI/PS and pH 1.0–2.0 for ZVA/PS) will be necessary. Further studies of 3,5-DCP and 2,4-DCA degradation focusing on the treatment of real (industrial) wastewater and investigation of aquatic toxicity (acute, chronic) could be useful to avoid the harmful, ecotoxicological undesired effects.

Supplementary Materials

## References

[ref1] (2014). Degradation of chlorophenols and alkylphenol ethoxylates, two representative textile chemicals, in water by advanced oxidation processes: the state of the art on transformation products and toxicity. Chemosphere.

[ref2] (2002). Preparation, characterization and photocatalytic activity of nanocrystalline thin film TiO2 catalysts towards 3,5-dichlorophenol degradation. Journal of Photochemistry and Photobiology A: Chemistry.

[ref3] (2003). -2,4-dichloroaniline in wheat (Triticum aestivum) and soybean (Glycine max). Uptake and metabolic fate of [14C]-2.

[ref4] (1977). Composition, treatment efficiency, and environmental significance of dye manufacturing plant effluents. Analytical Chemistry.

[ref5] (2019). An overview of chlorophenols as contaminants and their removal from wastewater by adsorption: a review. Journal of Environmental Management.

[ref6] (1998). Phototransformation of 2,4-dichloroaniline in a surface freshwater environment: effects on microbial assemblages. Bulletin of Environmental Contamination and Toxicology.

[ref7] (2005). Modeling of chlorophenols competitive adsorption on soils by means of the ideal adsorbed solution theory. Journal of Hazardous Materials.

[ref8] (1997). Chlorinated phenolic compounds in coniferous needles. Effects of metal and paper industry and incineration. Chemosphere.

[ref9] (2018). Linking mode of action of the model respiratory and photosynthesis uncoupler 3,5-dichlorophenol to adverse outcomes in Lemna minor. Aquatic Toxicology.

[ref10] (2017). The toxic effects of chlorophenols and associated mechanisms in fish. Aquatic Toxicology.

[ref11] (2003). Expression of delayed cell death (DCD) in the progeny of fish cells surviving 2,4-dichloroaniline (2,4-DCA) exposure. Aquatic Toxicology.

[ref12] (2003). Effects of ultrahigh dilutions of 3,5-dichlorophenol on the luminescence of the bacterium Vibrio fischeri. Biochimica et Biophysica Acta (BBA) - General Subjects.

[ref13] (1989). Development of a standardized reproduction toxicity test with the earthworm species Eisenia fetida andrei using copper, pentachlorophenol, and 2,4-dichloroaniline. Ecotoxicology and Environmental Safety.

[ref14] (2016). Effects of 3,5-dichlorophenol on excess biomass reduction and bacterial community dynamics in activated sludge as revealed by a polyphasic approach. Journal of Bioscience and Bioengineering.

[ref15] (2008). Characterization of the toxic effects of cadmium and 3.5-dichlorophenol on nitrifying activity and mortality in biologically activated sludge systems - effect of low temperature. Environmental Science and Pollution Research.

[ref16] (2000). Decision N0 2455/2001/EC of the European Parliament and of the Council of 20 November 2001. amending Directive.

[ref17] (1980). Agency (EPA). Ambient water quality criteria for chlorinated phenols. Rep.

[ref18] (2012). Transformation of 2,4-dichlorophenol by H_2_O2/UV-C, Fenton and photo-Fenton processes: oxidation products and toxicity evolution. Journal of Photochemistry and Photobiology A: Chemistry.

[ref19] (2017). Zero-valent aluminum for reductive removal of aqueous pollutants over a wide pH range: performance and mechanism especially at near-neutral pH. Water Research.

[ref20] (2019). –11 by peroxymonosulfate via controlling the reactive oxygen species over Ce substituted 3D Mn2O3. Enhanced.

[ref21] (2020). Investigation of iohexol degradation kinetics by using heat-activated persulfate. Chemical Engineering Journal.

[ref22] (2019). Ascorbic acid induced activation of persulfate for pentachlorophenol degradation. Chemosphere.

[ref23] (2009). Zero-valent aluminum for oxidative degradation of aqueous organic pollutants. Environmental Science & Technology.

[ref24] (2004). -dichlorophenol isomers within aqueous solutions. Ozonation of 2.

[ref25] (2018). Treatment of organic pollutants by homogeneous and heterogeneous Fenton reaction processes. Environmental Chemistry Letters.

[ref26] (2006). Assessment of photo-Fenton and biological treatment coupling for Diuron and Linuron removal from water. Water Research.

[ref27] (2019). Electrochemical degradation of sunscreen agent benzophenone-3 and its metabolite by Ti/SnO2-Sb/Ce-PbO2 anode: kinetics, mechanism, toxicity and energy consumption. Science of The Total Environment.

[ref28] (2017). Sonolysis and sono-Fenton oxidation for removal of ibuprofen in (waste)water. Ultrasonics Sonochemistry.

[ref29] (2017). Activation of peroxymonosulfate by microwave irradiation for degradation of organic contaminants. Chemical Engineering Journal.

[ref30] (2018). Evaluation of degradation and kinetics parameters of acid orange 7 through wet air oxidation process. The Canadian Journal of Chemical Engineering.

[ref31] (2018). Huang Q. Degradation of 2, 4-dichlorophenol in aqueous solution by dielectric barrier discharge: effects of plasma-working gases, degradation pathways and toxicity assessment. Chemosphere.

[ref32] (2018). Comparison of pharmaceutical abatement in various water matrices by conventional ozonation, peroxone (O3/H_2_O2), and an electro-peroxone process. Water Research.

[ref33] (1967). Pulse radiolysis of aqueous solutions of sulfuric acid. Bulletin of the Academy of Sciences of the USSR Division of Chemical Science.

[ref34] (1988). Rate constants for reactions of inorganic radicals in aqueous solution. Journal of Physical and Chemical Reference Data.

[ref35] (1988). Critical Review of rate constants for reactions of hydrated electrons, hydrogen atoms and hydroxyl radicals (⋅OH/⋅O- in aqueous solution. Journal of Physical and Chemical Reference Data.

[ref36] (2017). Comparison of the reactivity of ibuprofen with sulfate and hydroxyl radicals: an experimental and theoretical study. Science of The Total Environment.

[ref37] (2018). Removal of an X-Ray contrast chemical from tertiary treated wastewater: investigation of persulfate-mediated photochemical treatment systems. Catalysis Today.

[ref38] (2018). Transition metal catalyzed sulfite auto-oxidation systems for oxidative decontamination in waters: a state-of-the-art mini review. Chemical Engineering Journal.

[ref39] (2016). Persulfate and hydrogen peroxide-activated degradation of bisphenol A with nano-scale zero-valent iron and aluminum. Journal of Advanced Oxidation Technologies.

[ref40] (2018). Activation of persulfate by homogeneous and heterogeneous iron catalyst to degrade chlortetracycline in aqueous solution. Chemosphere.

[ref41] (2017). Zero-valent aluminum-mediated degradation of bisphenol A in the presence of common oxidants. Water Science and Technology.

[ref42] (2015). Treatment of aqueous bisphenol A using nano-sized zero-valent iron in the presence of hydrogen peroxide and persulfate oxidants. Water Science and Technology.

[ref43] (2016). Zero-valent iron-activated persulfate oxidation of a commercial alkyl phenol polyethoxylate. Environmental Technology.

[ref44] (2018). Iopamidol degradation with ZVI- and ZVA-activated chemical oxidation: investigation of toxicity, anaerobic inhibition and microbial communities. Journal of Environmental Chemical Engineering.

[ref45] (2016). Zero-valent iron (ZVI) activation of persulfate (PS) for oxidation of bentazon in water. Chemical Engineering Journal.

[ref46] (2019). Efficient degradation of imidacloprid in water through iron activated sodium persulfate. Chemical Engineering Journal.

[ref47] (2019). High-energy ball milling enhancing the reactivity of microscale zero-valent aluminum toward the activation of persulfate and the degradation of trichloroethylene. Chemical Engineering Journal.

[ref48] (2017). Removal of iopamidol, an iodinated X-ray contrast medium, by zero-valent aluminum-activated H_2_O2 and S_2_O_8_^2−^. Chemical Engineering Journal.

[ref49] (2007). Characterization and pillaring of a Turkish bentonite (Resadiye). Journal of Colloid and Interface Science.

[ref50] (2000). Sorption of organic compounds by Al and Zr-hydroxy-intercalated and pillared bentonite. Clays and Clay Minerals.

[ref51] (1992). Institute of Environmental Health Sciences, National Institutes of Health (NTP).

[ref52] (2014). Review of iron-free Fenton-like systems for activating H_2_O2 in advanced oxidation processes. Journal of Hazardous Materials.

[ref53] (1963). Colorimetric determination of persulfate with alcian blue. Analytica Chimica Acta.

[ref54] - Application of inductively coupled plasma mass spectrometry (ICP-. TS EN ISO 17294-2.

[ref55] (1989). Rate constants for hydrogen abstraction reactions of the sulfate radical, SO_4_^·-^. Alkanes and ethers. International Journal of Chemical Kinetic.

[ref56] (2004). Barker JR. The Journal of Physical Chemistry A.

[ref57] (2005). Optimization of Fenton process for the treatment of landfill leachate. Journal of Hazardous Materials.

[ref58] (2014). New insights into the role of zero-valent iron surface oxidation layers in persulfate oxidation of dibutyl phthalate solutions. Chemical Engineering Journal.

[ref59] (2015). -dichlorophenol using iron-based nanoparticles and persulfate system. Heterogeneous Fenton oxidation of 2.

[ref60] (2017). Oxidative degradation of Triton X-45 using zero valent aluminum in the presence of hydrogen peroxide, persulfate and peroxymonosulfate. Catalysis Today.

