# Supplementary material for: Treatment of industrial contaminants with zero-valent iron- and zero-valent aluminium-activated persulfate: a case study with 3,5-dichlorophenol and 2,4-dichloroaniline
Source: Turk J Chem. 2021 Apr 28;45(2):269–81. doi: 10.3906/kim-1911-60 (PMC8164209; doi:10.3906/kim-1911-60)
Supplement: Supplementary file 1 — Supplementary Materials [file turkjchem-45-269-sup001.pdf]

Supplementary material

Table S1. 3,5-DCP and 2,4-DCA abatements obtained during application of ZVA/air/H^+ and ZVI/air/H^+ treatment systems. Experimental conditions: 3,5-DCP and 2,4-DCA = 2.0 mg/L, ZVI and ZVA = 1 g/L.

	3,5-DCP		2,4-DCA	
Time (min)	ZVA, pH 3.0	ZVI, pH 5.0	ZVA, pH 3.0	ZVI, pH 5.0
0	0	0	0	0
2	0	0	0	0
5	5	0	2	0
10	1	0	0	0
20	2	0	1	0
40	7	0	0	1
60	7	0	0	2
80	9	0	7	0
100	18	0	8	0
120	22	0	8	4

Table S2. 3,5-DCP abatements obtained during application of the ZVI/PS treatment system. Experimental conditions: 3,5-DCP = 2 mg/L, ZVI = 1 g/L, pH = 5.0.

Time (min)	0.10 mM PS	0.25 mM PS	0.50 mM PS	0.75 mM PS	1.00 mM PS	0.5 mM PS pH 3.0
0	0	0	0	0	0	0
2	0	4	0	10	5	37
5	0	2	7	0	6	71
10	0	1	2	4	4	90
20	0	4	1	3	5	100
40	0	5	4	3	12	100
60	0	0	3	6	17	100
80	0	2	5	13	23	100
100	0	4	6	14	40	100
120	0	5	11	27	59	100

Table S3. 2,4-DCA abatements obtained during application of the ZVI/PS treatment system. Experimental conditions: 2,4-DCA = 2 mg/L, ZVI = 1 g/L, pH = 5.0.

Time (min)	0.10 mM PS	0.25 mM PS	0.50 mM PS	0.75 mM PS	1.00 mM PS	0.5 mM PS pH 3.0
0	0	0	0	0	0	0
2	13	6	0	11	0	51
5	7	5	4	16	0	88
10	15	6	7	10	0	100
20	12	3	9	13	10	100
40	15	6	18	19	18	100
60	16	9	33	45	28	100
80	13	18	49	100	65	100
100	12	18	72	100	100	100
120	15	23	91	100	100	100

Table S4. 3,5-DCP abatements obtained during application of the ZVA/PS treatment system. Experimental conditions: 3,5-DCP = 2 mg/L, ZVA = 1 g/L, pH = 3.0.

Time (min)	0.10 mM PS	0.25 mM PS	0.50 mM PS	0.75 mM PS	1.00 mM PS	0.5 mM PS pH 1.5
0	0	0	0	0	0	0
2	0	0	0	1	1	4
5	0	0	2	0	0	7
10	2	2	1	4	6	15
20	3	6	5	0	8	20
40	5	10	5	3	15	25
60	7	11	12	6	18	34
80	11	14	16	12	24	48
100	12	14	21	15	30	61
120	12	18	21	18	31	77

Table S5. 2,4-DCA abatements obtained during application of the ZVA/PS treatment system. Experimental conditions: 2,4-DCA = 2 mg/L, ZVA = 1 g/L, pH = 3.0.

Time (Min)	0.10 mM PS	0.25 mM PS	0.50 mM PS	0.75 mM PS	1.00 mM PS	0.50 mM PS, pH 1.5
0	0	0	0	0	0	0
2	9	0	0	3	8	7
5	7	4	0	5	4	11
10	9	3	0	12	6	16
20	13	10	6	10	5	24
40	12	21	5	15	9	33
60	15	28	12	16	14	43
80	19	29	13	21	22	58
100	21	46	17	26	25	77
120	22	47	20	33	29	89
